# Supplementary material for: Perceptions and priorities for the development of multiplex rapid diagnostic tests for acute non-malarial fever in rural South and Southeast Asia: An international modified e-Delphi survey
Source: PLoS Negl Trop Dis. 2022 Nov 11;16(11):e0010685. doi: 10.1371/journal.pntd.0010685 (PMC9683552; doi:10.1371/journal.pntd.0010685)
Supplement: S1 Appendix — (DOCX) [file pntd.0010685.s001.docx]

**S1 APPENDIX**

**Participants – Mainland Southeast Asia panel**

| **Name** | **Round 1** | **Round 2** |
| --- | --- | --- |
| Elizabeth Ashley | ✓ | ✓ |
| Stuart Blacksell |  | ✓ |
| Sotharith Bory | ✓ | ✓ |
| Yoke Fun Chan | ✓ | ✓ |
| Hock Hin Chua | ✓ | ✓ |
| John Crump | ✓ | ✓ |
| David Dance | ✓ | ✓ |
| Jittima Dhitavat |  | ✓ |
| Adeeba Kamarulzaman | ✓ | ✓ |
| Direk Limmathurotsakul | ✓ | ✓ |
| Joon Wah Mak | ✓ | ✓ |
| Mayfong Mayxay | ✓ | ✓ |
| Francois Nosten | ✓ | ✓ |
| Koukeo Phommasone | ✓ | ✓ |
| Aung Pyae Phyo | ✓ | ✓ |
| Punnee Pitisuttithum |  | ✓ |
| Paul Newton | ✓ | ✓ |
| Pratap Singhasivanon | ✓ | ✓ |
| Frank Smithuis | ✓ |  |
| Myo Swe |  | ✓ |
| Claudia Turner | ✓ | ✓ |
| Paul Turner | ✓ | ✓ |
| Timothy William | ✓ | ✓ |
| Muhamad Yazli Yuhana |  | ✓ |

**Participants – Maritime Southeast Asia panel**

| **Name** | **Round 1** | **Round 2** |
| --- | --- | --- |
| Josh Francis | ✓ | ✓ |
| Jemelyn García | ✓ |  |
| Charlotte Hall | ✓ | ✓ |
| Lois Hong-Rajaraman |  | ✓ |
| Jaime Montoya |  | ✓ |
| Erni Juwita Nelwan | ✓ | ✓ |
| Clare Nourse | ✓ | ✓ |
| Ric Price |  | ✓ |
| Kartika Saraswati | ✓ | ✓ |

**Participants – South Asia panel**

| **Name** | **Round 1** | **Round 2** |
| --- | --- | --- |
| Bipin Adhikari | ✓ | ✓ |
| Anup Bastola | ✓ | ✓ |
| Mohammad Abul Faiz | ✓ | ✓ |
| Razia Fatima |  | ✓ |
| Subhash Hira | ✓ | ✓ |
| Bushra Jamil | ✓ | ✓ |
| Abhilasha Karkey |  | ✓ |
| Summiya Nizamuddin | ✓ | ✓ |
| Christopher Parry | ✓ | ✓ |
| Firdausi Qadri | ✓ | ✓ |
| Priscilla Rupali | ✓ | ✓ |
| Naseem Salahuddin | ✓ | ✓ |
| Faisal Sultan | ✓ | ✓ |
