# Supplementary material for: Perceptions and priorities for the development of multiplex rapid diagnostic tests for acute non-malarial fever in rural South and Southeast Asia: An international modified e-Delphi survey
Source: PLoS Negl Trop Dis. 2022 Nov 11;16(11):e0010685. doi: 10.1371/journal.pntd.0010685 (PMC9683552; doi:10.1371/journal.pntd.0010685)
Supplement: Attachment — Submitted filename: Response to Reviewers.docx [file pntd.0010685.s002.docx]

Dr Rusheng Chew

Mahidol Oxford Tropical Medicine Research Unit

420/6 Ratchawithi Road

Ratchathewi 10400 Bangkok

Thailand

_____________________________________________________________________________________

Drs Shaden Kamhawi, Paul Bridley, and Peter Hotez

Editors-in-chief

PLoS Neglected Tropical Diseases

*Via email*	10 October 2022

______________________________________________

Dear Drs Kamhawi, Brindley, and Hotez

**Re: PNTD-D-22-00965 Perceptions and priorities for the development of multiplex rapid diagnostic tests for acute non-malarial fever in rural South and Southeast Asia: an international modified e-Delphi survey**

On behalf of all co-authors, many thanks for inviting us to revise and resubmit the abovementioned manuscript. We are glad to see that, on the whole, the reviews have been positive and thank the three reviewers for their time and effort.

We note that there are clear differences of opinion between Reviewer #1 and Reviewers #2 and #3. In responding to the reviewer comments, for each section of the manuscript we will first address the comments of the latter two, comparing and contrasting their remarks with those of the former where necessary. Line numbers quoted where describing changes made refer to those in the revised manuscript.

**Methods**

- ***Reviewer #2:***

*The objectives of the study are clearly articulated with a clear testable hypothesis stated.
The study design is appropriate to address the stated objectives.
The population is clearly described and appropriate for the hypothesis being tested.
The sample size is sufficient to ensure adequate power to address the hypothesis being tested.
Correct statistical analysis were used to support conclusions.
There are no concerns about ethical or regulatory requirements.*

- ***Reviewer #3:***

*This manuscript describes a study to obtain expert consensus on priority analytes which should be included in multiplex lateral flow rapid diagnostic tests (LF-RDTs) for patients with acute non-malarial fever in South and Southeast Asian LMICs. The authors conducted two rounds of a modified e-Delphi survey with 84 panellists from 12 countries in three regions, representing Mainland Southeast Asia, Maritime Southeast Asia, and South Asia. There was consensus that LF-RDTs should be incorporated into clinical decision support tools. At a minimum, these LF-RDTs should be able to diagnose dengue and enteric fever in all patient categories. There was a clear preference to develop LF-RDTs for pathogens not readily detected by existing technologies, and for direct diagnosis through antigen detection versus serologic methods. Pathogen biomarkers were prioritized over host inflammatory biomarkers, with CRP being the only host biomarker ranked consistently highly.

This is a well-designed and highly informative study. The objectives were clearly articulated and the study design is appropriate to address the study objectives. There are no ethical or regulatory concerns.*

We thank Reviewers #2 and #3 for their positive comments on our methods, particularly their appropriateness. No specific areas for revision were identified from their remarks.

- ***Reviewer #1:***

*The authors have attempted to develop a consensus through e-Delphi on prioritising analytes for inclusion in multiplex lateral flow rapid diagnostic tests (LF-RDTs) to diagnose non malarial fever in LMICs. The research question is relevant and important to address. Although the methodology is appropriate, the results lack any new insight or knowledge. The following are my specific comments:*

*1. After two rounds, they failed to achieve the prespecified consensus of 80%. The alternative strategy to add the most common responses does not seem to have given any meaningful insight. The result does not add any new knowledge.*

*2. The current common causes of acute febrile illness such as COVID-19 and influenza seem to have been totally overlooked.*

*3. A lack of clear results would raise the question of soundness of the methodology. How were the participants selected? Were the four scenarios used appropriate and pretested to avoid ambiguity?*

We thank Reviewer #1 for their critique, and are encouraged that they, like Reviewers #2 and #3 think our methodology is appropriate and the research question important.

Respectfully, however, we disagree that our findings do not provide any new insights or knowledge, for the following reasons. Firstly, given that there is no published research on such priority-setting targeted towards the South and Southeast Asian region, the results of any methodologically sound study (as all three reviewers think this is) would, by definition, add to the fund of knowledge on this topic. Secondly, not all Delphi surveys result in attainment of the pre-specified consensus thresholds, which are to some extent arbitrary, even if consensus-building is the primary goal of such exercises. In our opinion, a finding that there are quite divergent views but which are then systematically analyzed using a rigorous method, as we have done by weighting the ranked selections of the respondents, is just as important where insights are concerned. In fact, given the lacuna in the current evidence base, our findings are, indeed, new knowledge.

The perception that the current common causes of acute febrile illness, such as COVID-19 and influenza, were overlooked is not the case. As stated in the last paragraph of the Introduction (original manuscript, lines 110-112), the population of interest is this study “acutely febrile rural residents of South and Southeast Asian low- and middle-income countries (LMICs) who test negative for malaria and in whom the source of infection is unclear”. Both COVID-19 and influenza are, primarily, respiratory illnesses and, thus, would be expected to have symptoms localizing to the respiratory system as the source of infection. It was, thus, expected that respondents would rank these diseases lower, if at all, given that they are much less likely to present as a fever of unclear source. In keeping with this, while some respondents did rank these diseases, they did not feature in the top seven items in any rank list; this is a rather different conclusion than that these diseases were ‘overlooked’.

Finally, we are puzzled by Reviewer #1’s last comment that the methods may not have been sound, given that their first comment noted their appropriateness. In response, we again refer to the explanation given previously regarding non-achievement of pre-specified consensus thresholds and how this is not necessarily a methodological failure. We also wish to provide reassurance that we did, indeed, pre-test the questionnaires and that experts were selected based on pre-specified objective criteria, as stated in the Methods:

“Prior to launch, two experts who would have otherwise been invited as panellists were asked to review the survey to ensure face validity, readability, and usability.” (lines 119-121)

“Potential panelists were shortlisted through searches of national infectious diseases and/or tropical medicine specialty society office-bearers; leaders of health-related non-governmental organizations and governmental bodies; researchers from university departments or faculties; and recommendations of experts already selected. The final list was constructed based on work experience, expertise and reputation, involvement in health policymaking, and publication record. To balance perspectives, at least 30% of each regional panel were in non-clinical roles and at least 25% were women.” (lines 132-137)

**Results**

- ***Reviewer #2:***

*The analysis presented match the analysis plan.*

*The results are clearly and completely presented.*

*The figures (Tables and Images) are of sufficient quality for clarity.*

- ***Reviewer #3:***

*The analysis presented matched the analysis plan. The results are visually presented as a series of graphs that are easy to understand, despite their complexity. The figures are of sufficient quality for clarity.*

Again, we thank Reviewers #2 and #3 for their positive reviews. No specific areas for revision were identified from their remarks.

- ***Reviewer #1:***

*4. The findings could have been better presented. Figures 1, 2 and 3 could have been represented in a tabular format with percentages for a quick review as it’s difficult to read the graphs with too many colours. Figure 5 is well presented but it would help to add data labels (%) to each of the bars for better understanding and clarity.*

We thank Reviewer #1 for their comments, and have revised Figure 5 to include data labels to increase its clarity. However, Figures 1-3 are not appropriate for presentation in the manner suggested since they are constructed from rank lists with each item being weighted, rather than simple numbers and proportions. Presenting such data as tables would make the tables long, unwieldy, and even harder to comprehend quickly. In view of this, as well as the comments by Reviewers #2 and #3 that the figures are clear and easy to understand, we have elected not to revise Figures 1-3.

**Conclusions**

- ***Reviewer #2:***

*The conclusions are supported by the data presented.*

*The limitations of analysis are clearly described.*

*The authors discuss adequately, precisely and clearly how these data can be helpful to advance our understanding of the topic under study.*

*Public health relevance is addressed.*

- ***Reviewer #3:***

*The conclusions are consistent with the findings of the study. The limitations of the study are clearly stated.*

We thank Reviewers #2 and #3 for their positive assessments of our conclusions. Reviewer #1 did not provide a response. As no recommendations for revision were received, we have not made any changes to this section.

**Editorial and data presentation modifications**

- ***Reviewer #2:***

*This paper represents an international modified e-Delphi survey that deals with the perceptions and priorities of selected infectious disease experts, who live and work in the area of South and Southeast Asia, for the development of acute non-malarial fever multiplex rapid diagnostic tests. The results of this study provide a valuable source of data on pathogen-specific biomarker analytes that should be incorporated in the test which would contribute significantly to rapid differential diagnosis of potential etiologic agents of acute non-malarial fever in resource-poor settings of above mentioned regions. In addition, the manuscript is clearly written, easy to read and understand. Given that there is very little data on this problem in the current literature, this study deserves to be published in “PLOS Neglected Tropical Diseases” journal. This reviewer has no additional suggestions.*

We thank Reviewer #2 for these very encouraging comments; Reviewer #3 did not provide a response for this section. We did not identify any areas for revision from these comments, but wish to reiterate that Reviewer #2, like us, in these comments feel that the paucity of published data on this subject is a clear impetus for publication of our results in this journal.

- ***Reviewer #1:***

*The introduction does not have a logical flow of thought. The opening statement of ‘Fever is a common but non-specific sign of sepsis’ itself is misleading. Sepsis commonly occurs secondary to a bacteraemic illness from a focus of infection such as pyelonephritis, pneumonia, intra-abdominal source, cellulitis, skin and soft tissue infection etc. The LF-RDTs are targeted towards tropical infections without a clear focus such as dengue, typhoid, scrub typhus / rickettsial infections, leptospirosis etc. The first paragraph confuses the goal with ‘sepsis’. Another example of lack of clarity is the sentence starting with Line101 which states ‘In addition, there is growing interest in augmenting pathogen-based diagnostics by assaying host biomarkers of inflammation in parallel, in particular those which can assist in the differentiation viral from non-viral infections, thus improving antimicrobial stewardship,[13] or predict or indicate severe disease, thus improving the identification of patients who require* *escalation of care.[14]’. Combining many ideas is confusing and made it very complex. The introduction requires substantial modification.*

We thank Reviewer #1 for their suggestions on how the clarity of the Introduction can be improved. We wish, firstly, to state that while respondents felt that the ‘tropical infections without a clear focus’ should be prioritized for potential LF-RDTs in accordance with the survey aims and target patient population, these infections are not mutually exclusive with the other syndromes quoted by Reviewer #1 in terms of their potential to cause sepsis, as demonstrated, for example, by these publications:

- Thap LC, et al. Septic shock secondary to scrub typhus: characteristics and complications. (2002) Southeast Asian J Trop Med Public Health. 33(4):780-6
- Subedi P, et al. (2021) Dengue and scrub typhus co-infection causing septic shock. Oxf Med Case Reports. 2021(11):omab115.
- Ugas MB, et al. (2016) *Salmonella* Typhi-Induced Septic Shock and Acute Respiratory Distress Syndrome in a Previously Healthy Teenage Patient Treated With High-Dose Dexamethasone. J Investig Med High Impact Case Rep. 4(2):2324709616652642.

Nevertheless, to avoid any confusion, we have revised the first sentence to read as follows:

“Infection, which may lead to sepsis, commonly presents with fever.[1]” (line 1)

With regard to Reviewer #1’s other comment that ‘combining many ideas is confusing and made it very complex’, we respectfully disagree. Firstly, only two other concepts crucial to the development of potential LF-RDTs were featured in the survey i.e., prognostication and pathogen class differentiation. These are important issues to address through a rapid point-of-care test, because for low- or partially-skilled health workers in resource-poor settings, they help answer the questions of whether a patient should be referred to higher-level care (which may be distant and costly, leading to a drain on resources on both the part of the healthcare system and patient) or prescribed antimicrobials, if a viral infection can be reliably predicted in the absence of a positive pathogen-based diagnosis (thus targeting antimicrobials appropriately). We are of the opinion that where questions of diagnosis, prognosis, and management can be addressed rapidly, simultaneously, and economically, we must attempt to do so, not just in the interests of good patient care but also good resource allocation.

However, we are grateful for Reviewer #1’s observation that the readability of the sentence in line 101 of the original manuscript can be improved. We have, therefore, split it into three shorter sentences so the point made comes across more clearly:

“In addition, there is growing interest in augmenting pathogen-based diagnostics by assaying host biomarkers of inflammation in parallel. These biomarkers can be broadly categorized by use-case. The first of these use-cases is differentiation of viral from non-viral infections, thus improving antimicrobial stewardship.[13] The second is prediction or indication of severe disease, thus improving the identification of patients who require escalation of care.[14]

**Summary and general comments**

- ***Reviewer #3:***

*Identification of regional priorities for clinical support of major syndromes is an important first step but studies have shown that availability of a diagnostic test is no guarantee that it will be used. This may be outside of the scope of this survey but it is important that the design of LF-RDTs for these priorities needs to take into account factors that will allow the tests to be widely deployed. If the authors have included in their survey questions related to the usability or challenges with current RDTs, it would be very useful to include them in the manuscript. Some examples for consideration by the authors are:*

*1. LF-RDTs for dengue IgM and NS1 are already commercially available. Since RDTs for dengue appear to be highly ranked in terms of priority, did the authors ask why they are not widely used? Do they need to have better performance or do they need to be multiplex to be useful (I.e. include other causes of febrile illness)?*

*2. Rapid tests for CRP have been available free of charge in some developed countries, such as UK, but uptake is less than 30%. Reasons for this have not been well studied but appear to be related largely to the difficulty of performing the test within the current patient pathway or requiring an additional follow-up visit. Did the authors include questions on how CRPs are currently used in either the public or private sector within the three regions?*

*3. Major causes of fever may vary by season and geographic location, even within the 3 regions of this study. This will affect not only the design of the multiplex LF-RDTs but also interpretation of the test results in terms of predictive values and likelihood ratios. It will be useful to include such data, if available.*

We thank Reviewer #3 for these insightful comments; Reviewer #2 did not provide a response for this section. In the interest of keeping the survey questionnaire succinct and targeted (which, in turn, encourages completion), we did not include questions on usability or challenges with current RDTs. We do, however, fully agree that these questions are important and do need to be studied in the context of each intended setting before being deployed. We have, therefore, added the following suggestion for future research to the Discussion to reflect the insights raised by Reviewer #3:

“The last concerns implementation research on contextual and other factors influencing the uptake of commercially available non-multiplex RDTs whose analytes were included in the rank lists produced through this study. It is essential to understand the reasons why CRP and dengue RDTs, for example, are not used as widely as would be expected and why variations in uptake between settings exist,[31] because these may also be applicable to any multiplex LF-RDTs developed. It may also be the case that multiplexing may address some of the issues which have led to low uptake of single-plex assays, lending further support for multiplex LF-RDT development.” (lines 338-345)

We also concur with Reviewer #3 that spatial and temporal variation in disease epidemiology will affect the interpretation of test results. Keeping this in mind, but also being aware of the need for questionnaire brevity and the need for simplicity when the target end-users are under-skilled, we phrased the questions such that respondents selected analytes that would be useful year-round in their respective regions, with their perceptions on the usefulness of these tests also assessed through questions explicitly stating that they had sensitivities and specificities of 75% and 90%, respectively:

“… participants were provided with four scenarios in which they were required to rank seven analytes from a list of pathogen-specific and host inflammatory biomarkers for hypothetical LF-RDTs for acute (≤14 days duration) non-malarial fever of unclear source to be developed for year-round use in each region.” (lines 144-147)

“participants were asked whether they would find such a test with a minimum sensitivity of 75% and minimum specificity of 90% across all targets helpful to guide clinical decision-making…” (lines 164-166)

- ***Reviewer #1:***

*The authors have attempted to develop a consensus through e-Delphi on prioritising analytes for inclusion in multiplex lateral flow rapid diagnostic tests (LF-RDTs) to diagnose non malarial fever in LMICs. The research question is relevant and important to address. Although the methodology is appropriate, the results lack any new insight or knowledge.*

We again thank Reviewer #1 for these comments, which are similar to the ones we have already addressed in the Methods section above.

Thank you again for considering our revised manuscript; we look forward to a positive response in due course.

Yours sincerely

**Rusheng Chew**

On behalf of the authors
