# Supplementary material for: Perceptions and priorities for the development of multiplex rapid diagnostic tests for acute non-malarial fever in rural South and Southeast Asia: An international modified e-Delphi survey
Source: PLoS Negl Trop Dis. 2022 Nov 11;16(11):e0010685. doi: 10.1371/journal.pntd.0010685 (PMC9683552; doi:10.1371/journal.pntd.0010685)
Supplement: Attachment — Submitted filename: Response to Editors.docx [file pntd.0010685.s003.docx]

Dr Rusheng Chew

Mahidol Oxford Tropical Medicine Research Unit

420/6 Ratchawithi Road

Ratchathewi 10400 Bangkok

Thailand

_____________________________________________________________________________________

Drs Shaden Kamhawi, Paul Bridley, and Peter Hotez

Editors-in-chief

PLoS Neglected Tropical Diseases

*Via email*	28 October 2022

______________________________________________

Dear Drs Kamhawi, Brindley, and Hotez

**Re: PNTD-D-22-00965.R1 Perceptions and priorities for the development of multiplex rapid diagnostic tests for acute non-malarial fever in rural South and Southeast Asia: an international modified e-Delphi survey**

On behalf of my co-authors, I thank you for asking us to make a minor revision to the abovementioned article. Thank you also for the editorial recommendation below:

*Only a minor change is needed, the rest of the responses are satisfying: Please further modify the statement on sepsis, even better omit it as a term. The manuscript is powerful enough to not need underlining of its significance with, strained I believe, correlations with sepsis. Afterwards it will be immediately accepted*

We have accepted your suggestion in full and have, therefore, deleted any mention of sepsis from the article. The relevant paragraph in the Introduction now reads as follows (lines 70-77):

“Infection commonly presents with fever,[1] and in regions with high burdens of infectious diseases such as South and Southeast Asia, many patients present acutely with fever but without localising signs and symptoms. It was previously assumed that malaria was the cause in most patients presenting with such fevers, but the roll-out of highly accurate malaria rapid diagnostic tests along with the success of public health efforts to eradicate malaria has resulted in a large decline in malaria incidence.[2, 3] Febrile illnesses, however, are still a common cause for seeking medical care in rural communities and, while early intervention is crucial in the management of infection, obtaining clinical and/or microbiological diagnoses is difficult in these resource-poor settings.”

We hope this minor revision is to your satisfaction, and look forward to seeing our manuscript in print very soon.

Yours sincerely

**Rusheng Chew**

On behalf of the authors
